# Supplementary material for: The molecular make up of smoldering myeloma highlights the evolutionary pathways leading to multiple myeloma
Source: Nat Commun. 2021 Jan 12;12:293. doi: 10.1038/s41467-020-20524-2 (PMC7804406; doi:10.1038/s41467-020-20524-2)

Supplemental: The Molecular Make Up of Smoldering Myeloma Highlights the Evolutionary Pathways Leading to Multiple Myeloma

Supplementary Material

Eileen M Boyle[1,2,3], Shayu Deshpande[1], Ruslana Tytarenko[1], Cody Ashby[1,4], Yan Wang[1], Michael A Bauer[1,4], Sarah K Johnson[1], Christopher P Wardell[1,4], Sharmilan Thanendrarajan[1], Maurizio Zangari[1], Thierry Facon[5], Charles Dumontet[2], Bart Barlogie[6], Arnaldo Arbini[3], Even H. Rustad[3], Francesco Maura[7], Ola Landgren[7], Fenghuang Zhan[1], Frits van Rhee[1], Carolina Schinke[1],Faith E Davies[3], Gareth J Morgan[3*], Brian A Walker[8*]

[1] Myeloma Center, University of Arkansas for Medical Sciences, Little Rock, AR, USA
[2] INSERM 1052/CNRS 5286 Cancer Research Center of Lyon, Lyon, FRANCE
[3] Perlmutter Cancer Center, NYU Langone Health, New York, NY, USA
[4] Department of Biomedical Informatics, University of Arkansas for Medical Sciences, Little Rock, AR, USA
[5] Service des maladies du sang. Hôpital Claude Huriez, Lille University Hospital, Lille, FRANCE.
[6] Division of Hematology, The Mount Sinai Hospital, New York, NY, USA
[7] Myeloma Service, Department of Medicine, Memorial Sloan Kettering Cancer Center, New York, NY, USA.
[8] Division of Hematology Oncology, Indiana University, Indianapolis, IN, USA.

*These authors contributed equally.

Supplemental Table 1: The incidence of copy number changes in SMM (n=82) and MM (n=223) based on CN estimates from the targeted panel (X^2=chi-squared statistic, two-sided *p*-value derived from Kruskal Wallis test).

	**MM**	**SMM**	X^2	***p*-value**
**gain(1q)**	33.63%	26.83%	0.9	0.3
**amp(1q)**	5.38%	4.88%	0.01	0.9
**del(11q)**	4.93%	3.66%	0.2	0.6
**Trisomy 11**	49.33%	40.24%	1.9	0.1
**del(1p): *CDKN2C***	17.04%	2.44%	11.2	0.0008
**Trisomy 9**	56.95%	46.34%	2.7	0.12
**del(16q): *CYLD***	26.01%	12.20%	5.8	0.01
**del(2p)**	2.44%	4.48%	0.05	0.8
**del(1p): *FAM46C***	22.87%	8.54%	7.8	0.008
**Trisomy 6**	21.52%	18.29%	0.2	0.6
**del(16q): *MAF***	28.25%	13.41%	6.3	0.01
**del(6q)**	15.70%	13.41%	0.06	0.2
**del(13q)**	48.43%	41.46%	0.9	0.3
**del(17p)**	15.70%	6.10%	4	0.04
**del(14q)**	19.28%	7.32%	5.5	0.02

Supplemental Table 2: Summary of sample sequencing metrics (n=number of patients).

	Translocation panel Depth	Mutation panel Depth
**SMM (n=82)**	363 (332-394)	786 (692-867)
**EM (n=10)**	357 (321-406)	735 (731-891)
**MGUS (n=17)**	413 (340-433)	974 (834-1036)

Supplemental Table 3: List of genes on the targeted panel. In bold are the previously described mutational driver genes.

ARID1A	***CHD2***	***FBXW7***	*KRAS*	***PSMG2***
ARHGEF12	*CHD4*	*FCHSD2*	*LRP1B*	***PTPN11***
ARID2	*CHEK1*	***FGFR3***	*LRRK2*	*RAD50*
ASXL1	*CHEK2*	*HDAC1*	***LTB***	***RB1***
ATM	*CRBN*	*HDAC4*	***MAF***	*RBX1*
ATR	***CREBBP***	*HDAC7*	***MAFB***	***SETD2***
ATRX	*CUL4A*	*HIST1H1C*	*MAP3K14*	***SF3B1***
BCL10	*CUL4B*	*HIST1H1D*	***MAX***	*SMARCA4*
BCL6	*CXCR4*	***HIST1H1E***	*MKI67*	*STAT3*
BCL7A	***CYLD***	***IDH1***	*MLL*	*TAF1*
BCORL1	*DDB1*	***IDH2***	*MYC*	*TET1*
BIRC2	***DIS3***	*IKZF1*	*MYD88*	***TET2***
BIRC3	***DNMT3A***	*IKZF3*	*NCKAP5*	*TET3*
BRAF	*DOT1L*	*IKZF4*	***NCOR1***	***TP53***
BRCA1	*EGFR*	***IRF4***	*NEDD9*	***TRAF2***
BRCA2	***EGR1***	*JAK1*	***NF1***	***TRAF3***
BRD4	***EP300***	*JAK2*	*NOTCH1*	*U2AF1*
BRF1	*EZH1*	*JAK3*	*NOTCH4*	*VSIG6*
CARD11	*EZH2*	*KAT6A*	*NR3C1*	*WHSC1*
CCND1	*FAF1*	*KDM2B*	***NRAS***	*WHSC1L1*
CCND3	***FAM46C***	*KDM5A*	*PCLO*	***XBP1***
CD36	*FANCA*	***KDM6A***	*POT1*	*ZFHX4*
CDKN1B	*FANCD2*	***KMT2B***	***PRDM1***	*ZRSR2*
CDKN2C	*FANCI*	***KMT2C***	***PRKD2***	
CHD1	*FANCM*	*KMT2D*	*PSMB5*	

Supplemental Table 4: Metrics of sequential samples.

Name	Median depth	Purity by flow	PYCLONE
A_2	99	74	Yes
A_3	88	70	Yes
A_4	89	90.4	Yes
A_5	86	83.4	Yes
A_6	103	91.26	Yes
B_2	85	94	Yes
B_3	110	98	Yes
B_6	116	67	Yes
B_7	103	87.1	Yes
B_8	106	64	Yes
C_1	90	97.9	Yes
C_2	93	94.7	Yes
C_4	105	89	Yes
C_5	89	85	Yes
C_6	92	80	Yes
C_7	90	60	Yes
C_8	100	80.2	Yes
D_3	126	86	Yes
D_4	154	89	Yes
D_5	128	96	Yes
D_6	100	100	Yes
D_7	98	97	Yes
E_10	80	99.6	Yes
E_2	151	85.3	Yes
E_3	98	84	Yes
E_4	108	91	Yes
E_5	123	87.9	Yes
E_6	115	94.3	Yes
E_7	103	87	Yes
E_8	91	87.6	Yes
E_9	90	98.7	Yes
F_2	112	85.9	Yes
F_3	90	87.4	Yes
F_4	77	90	Yes
F_5	96	89	Yes
F_6	91	68	Yes
F_7	104	100	Yes
F_8	91	94.2	Yes
G_1	81	91	Yes
G_2	99	92	Yes
H_1	99	60	No
H_10	68	98	No
H_2	70	80	No
H_5	81	90	No
H_6	81	97.5	No
H_8	76	99.1	No
H_9	68	98.5	No
I_1	78	96	Yes
I_4	106	87.6	Yes
I_5	85	92	No
I_6	89	95	No
I_8	108	99.5	Yes
I_9	84	99.4	Yes

Supplemental Table 5. ddPCR primer and probe sequences.

	***IGHG3-MYC* (5'-3')**	**non-translocated *IGH* locus (5'-3')**
**Forward primer**	CAGTATTTTAGTAGCTCAAAGACACCTCTT	AGCTGCCACCTGCTTGT
**Reverse primer**	GCTTAGGTCAGTTTTGCCCATCT	CTGGGCTGGGCTGAGTT
**Probes**	FAM-TCCATTTCTGAAGACTTA-MGBNFQ<br>VIC-AGTCCATTTCTGATGACTTA-MGBNFQ	FAM- TCCATTTCTGAAGACTTA-MGBNFQ<br>VIC- AGTCCATTTCTGATGACTTA-MGBNFQ

	Genes/Loci	Ch	Genes/Loci	Chr	Genes/Loci	Chr	Genes/Loci	Chr	Genes/Loci	Chr	Genes/Loci
**1**	*1pTEL*	**5**	*5q12*	**8**	*WHSC1L1*	**11**	*11qCEN*	**14**	*PSMB5*	**19**	*DOT1L*
	ARID1A		*CHD1*		*8pCEN*		*DDB1*		*FANCM*		*SMARCA4*
	HDAC1		*RAD50*		*KAT6A*		*CCND1*		*MAX*		*BRD4*
	FAF1		*EGR1*		*ZFHX4*		*FCHSD2*		*TRAF3*		*JAK3*
	CDKN2C		*NR3C1*		*NSMCE2*		*BIRC3*		*BRF1*		*19pCEN*
	JAK1		*5q32*		*TRIB1*		*BIRC2*	**15**	*VSIG6*		*KMT2B*
	BCL10	**6**	*IRF4*		*LINC00861*		*ATM*		*15qCEN*		*PRKD2*
	NRAS		*NEDD9*		*FAM84B*		*MLL*		*FANCI*		*19qTEL*
	1pCEN		*HIST1H1C*		*PCAT1*		*ARHGEF12*		*IDH2*	**20**	*ASXL1*
	FAM46C		*HIST1H1E*		*POU5F1B*		*CHEK1*		*CHD2*		*MAFB*
	1q21.3		*HIST1H1D*		*LOC727677*		*11qTEL*	**16**	*CREBBP*	**21**	*U2AF1*
	1qTEL		*LTB*		*MYC*	**12**	*KDM5A*		*16qCEN*	**22**	*CHEK2*
**2**	*DNMT3A*		*NOTCH4*		*PVT1*		*CD27*		*CYLD*		*XBP1*
	ALKi19		*CCND3*		*LOC728724*		*CHD4*		*MAF*		*RBX1*
	TET3		*6qCEN*		*GSDMC*		*CDKN1B*		*16qTEL*		*EP300*
	NCKAP5		*PRDM1*		*8qCEN*		*KRAS*		*FANCA*	**23**	*ZRSR2*
	CXCR4		*PARK2*	**9**	*9pTEL*		*12pCEN*	**17**	*17pTEL*		*KDM6A*
	LRP1B	**7**	*CARD11*		*JAK2*		*LRRK2*		*TP53CN*		*TAF1*
	SF3B1		*IKZF1*		*CDKN2A*		*ARID2*		*TP53*		*ATRX*
	IDH1		*EGFR*		*CDKN2B*		*HDAC7*		*NCOR1*		*CUL4B*
	HDAC4		*7pCEN*		*9pCEN*		*KMT2D*		*17pCEN*		*BCORL1*
**3**	*CRBN*		*7qCEN*		*NOTCH1*		*IKZF4*		*NF1*		
	FANCD2		*CD36*		*TRAF2*		*PTPN11*		*IKZF3*		
	MYD88		*PCLO*	**10**	*TET1*		*KDM2B*		*STAT3*		
	SETD2		*POT1*		*MKI67*		*BCL7A*		*EZH1*		
	3p		*BRAF*			**13**	*13qCEN*		*BRCA1*		
	3q		*EZH2*				*BRCA2*		*MAP3K14*		
	ATR		*KMT2C*				*RB1*	**18**	*PSMG2*		
	BCL6						*DIS3*				
**4**	*FGFR3*						*13qTEL*				
	WHSC1						*CUL4A*				
	TET2										
	FBXW7										

Supplemental Table 6: List of loci included in the plot comparing SMM and MM copy number changes (Figure 1c)

Supplemental Figure 1: The number of mutations per sample identified on the targeted panel is lower in SMM than in MM. The Kruskal-Wallis test results at the top represents the overall test n=number of patients, two-sided p-value derived from Kruskal Wallis test. Boxplot representing second quartile, median, and third quartile, whiskers representing first and forth quartile.

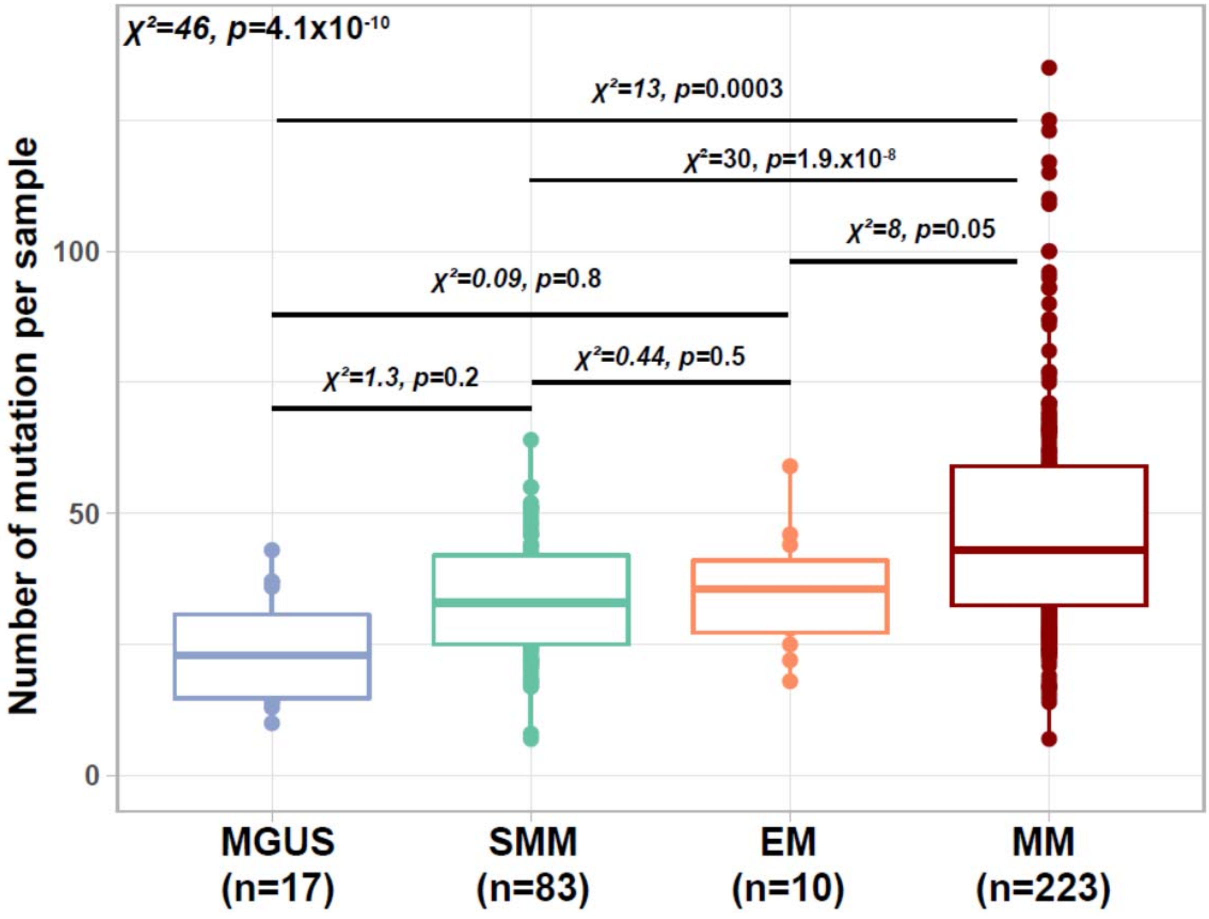

Supplemental Figure 2: Driver mutations increase with disease stages from MGUS to MM (MM=223, EM=10, SMM=82, and MGUS=17).

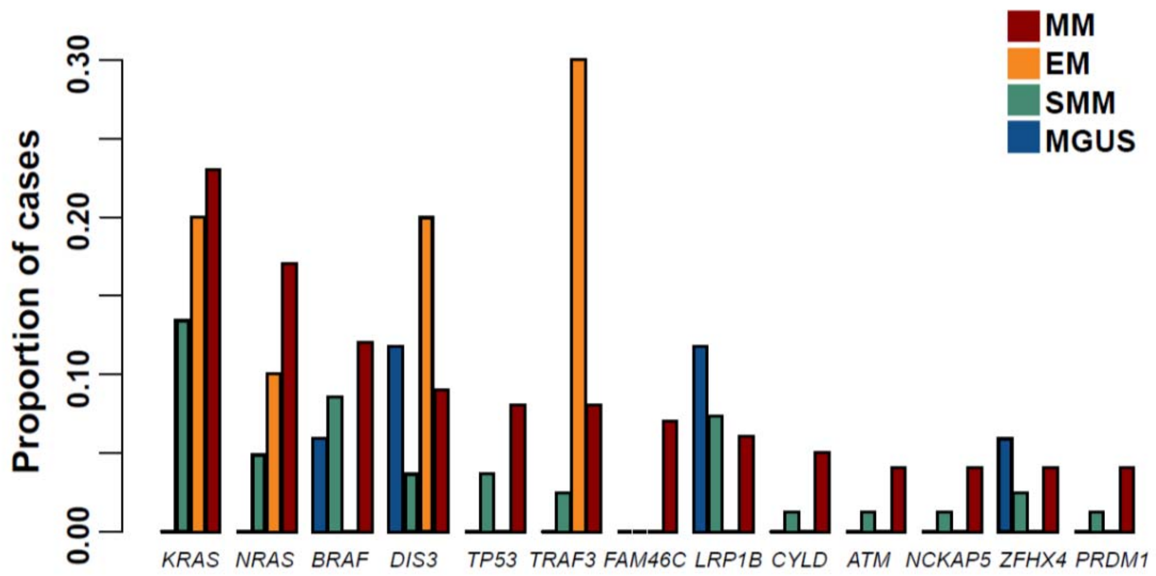

Supplemental Figure 3: Signature analysis a. Dendrogram representing the signatures present showing clustering of maf subgroup MM samples. b. There was a trend suggesting SMM patients with>5% APOBEC progressed faster than the others

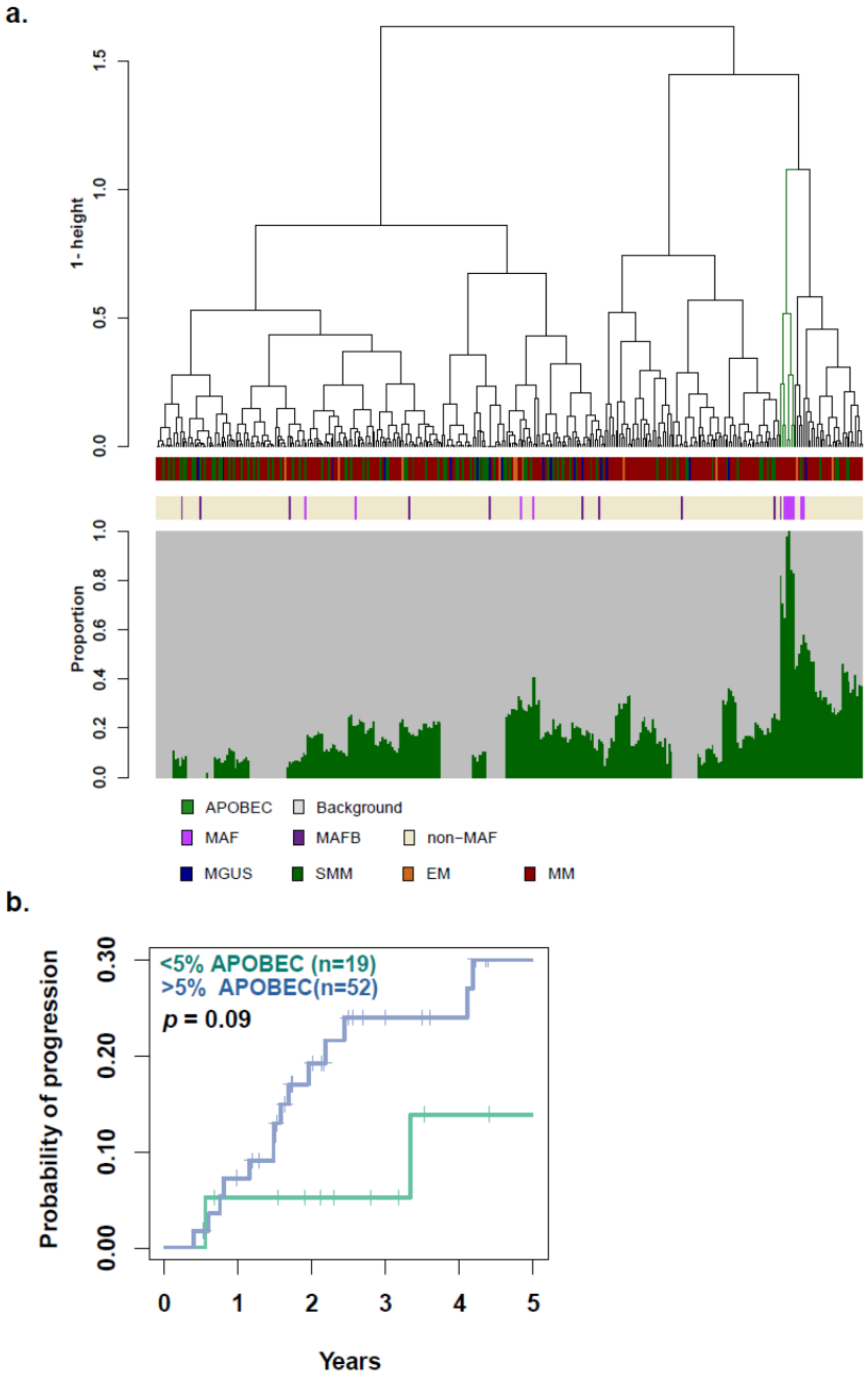

Supplemental Figure 4: Distribution of mutations per sample and risk group (IMWG).

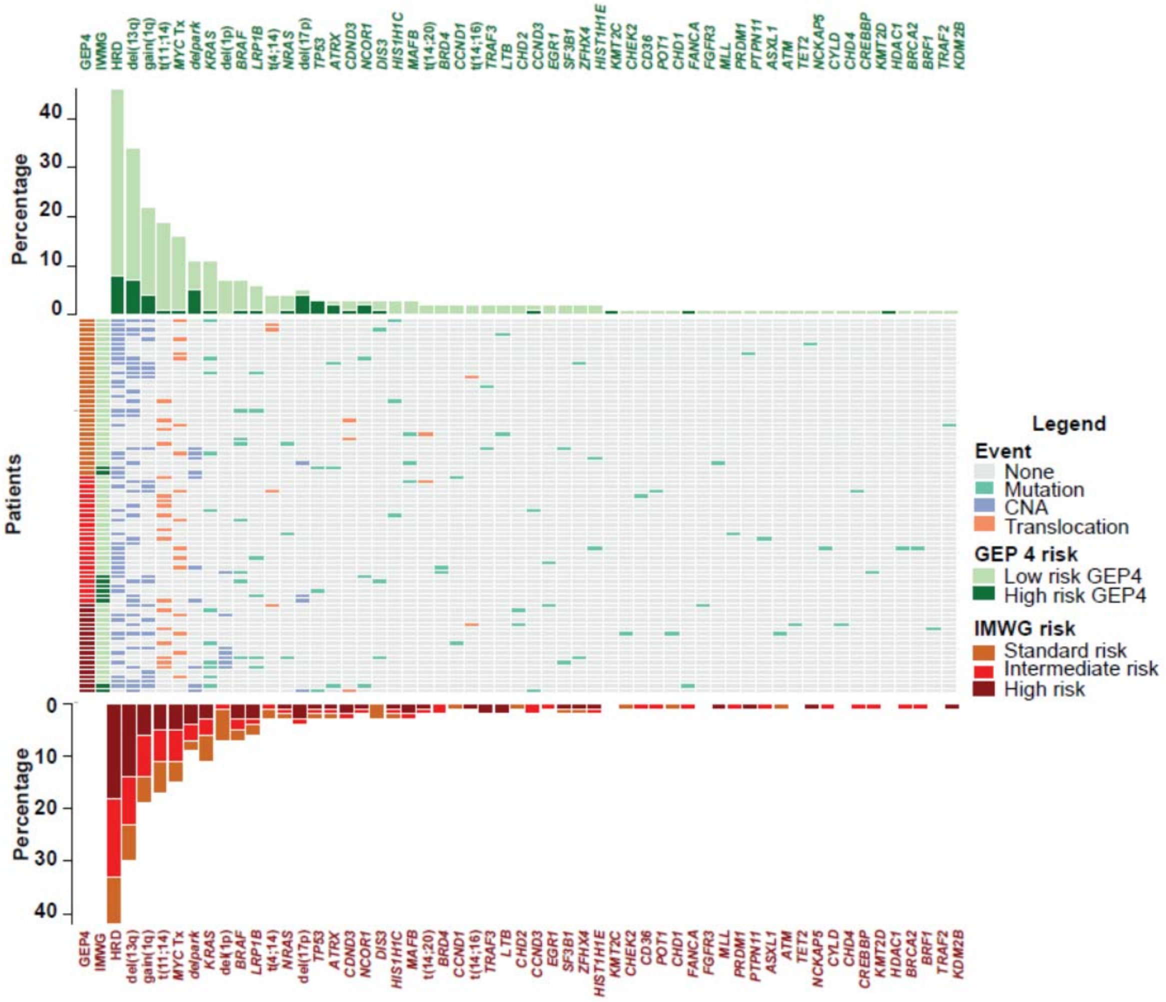

Supplemental Figure 5: Impact on progression free survival of a. del(6q) b. IMWG subgroups. c. GEP4 risk score. d. RAS (*BRAF/NRAS/KRAS*) mutations combined e. Multivariate analysis performed using all factors with an event present in n≥7 (IMWG, GEP4, del(6q), del(13q), mut/del *TP53*, and *KRAS* mutations). n=number of patients with the mutation and N=total number of patients evaluated, error-bars=95% CI, *p*= logrank test.

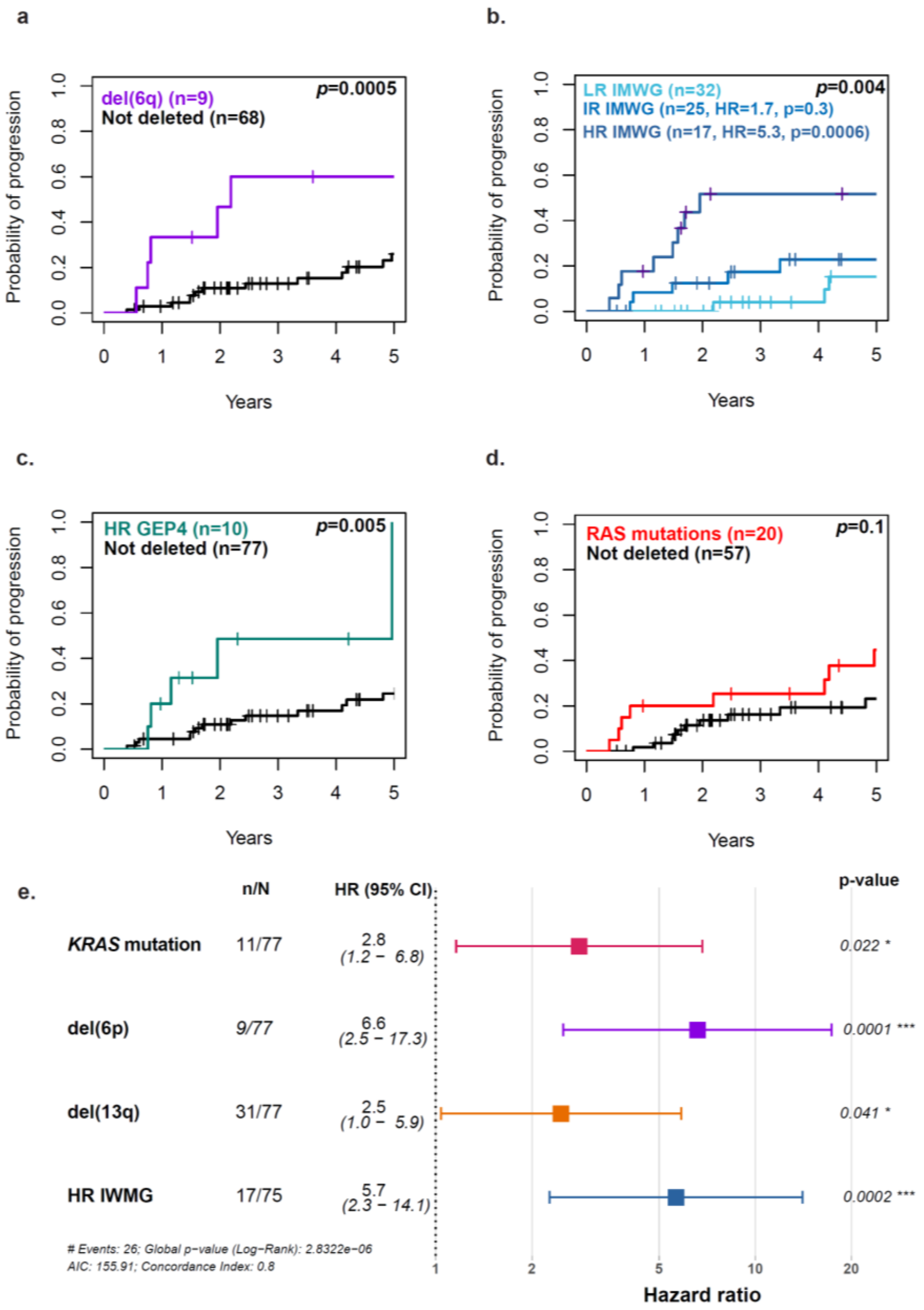

Supplemental Figure 6: Analysis of the patients that were within 3 months from initial diagnosis suggesting a. *KRAS* mutations have a stronger impact on progression among the ND SMM patients. b. GEP4, *KRAS* mutations, and HR mutations segregate patients effectively *p*= logrank test.

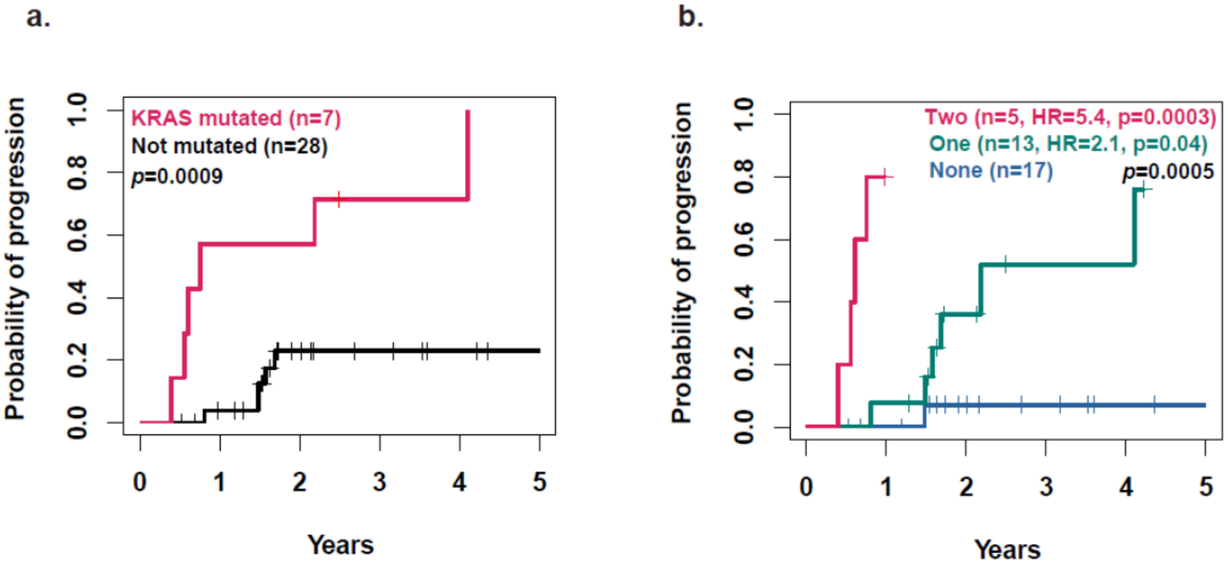

Supplemental Figure 7: NF-κB score in SMM is similar to MM and not MGUS. The Kruskal-Wallis test results at the bottom represents the overall test. n=number of patients, two-sided *p*-value derived from Kruskal Wallis test.

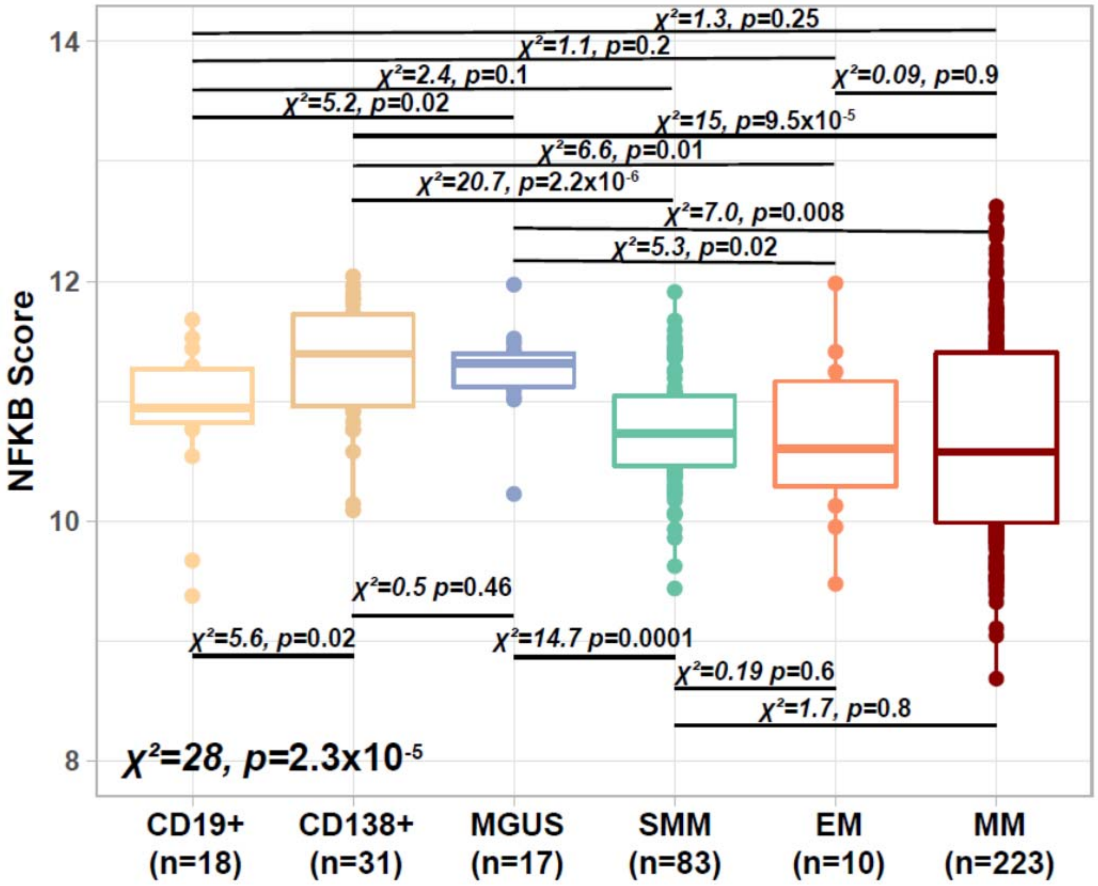

Supplemental Figure 8: Evolution of CNA events in sequential sample. Highlighted in green and red are the changing losses and gains, respectively.

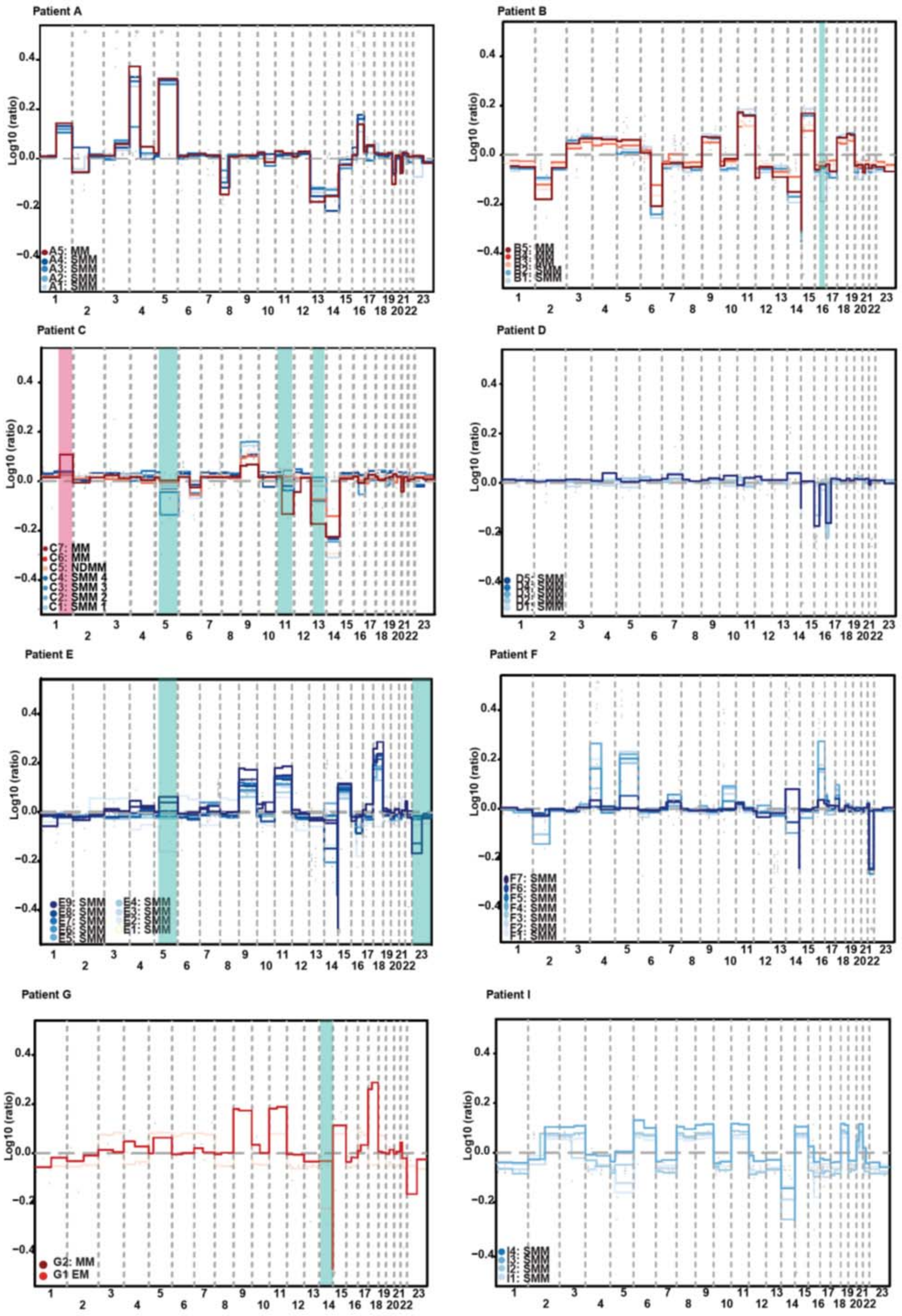

Supplemental Figure 9: Subgroup analysis of number of mutations per sample over time. a. Mutation rate over time in HRD vs nHRD. b. Mutation rate in progressors vs non progressors. c. Number of mutations per sample between SMM and MM. d. Number of drivers per sample between SMM and MM. χ^2=chi-statistic, r^2=coefficient of determination, Error bands=95%CI, p=one-way ANOVA (a-b) two-sided p-value derived from Kruskal-Wallis test (c-d), n=number of samples. Boxplot representing second quartile, median, and third quartile, whiskers representing first and last quartile.

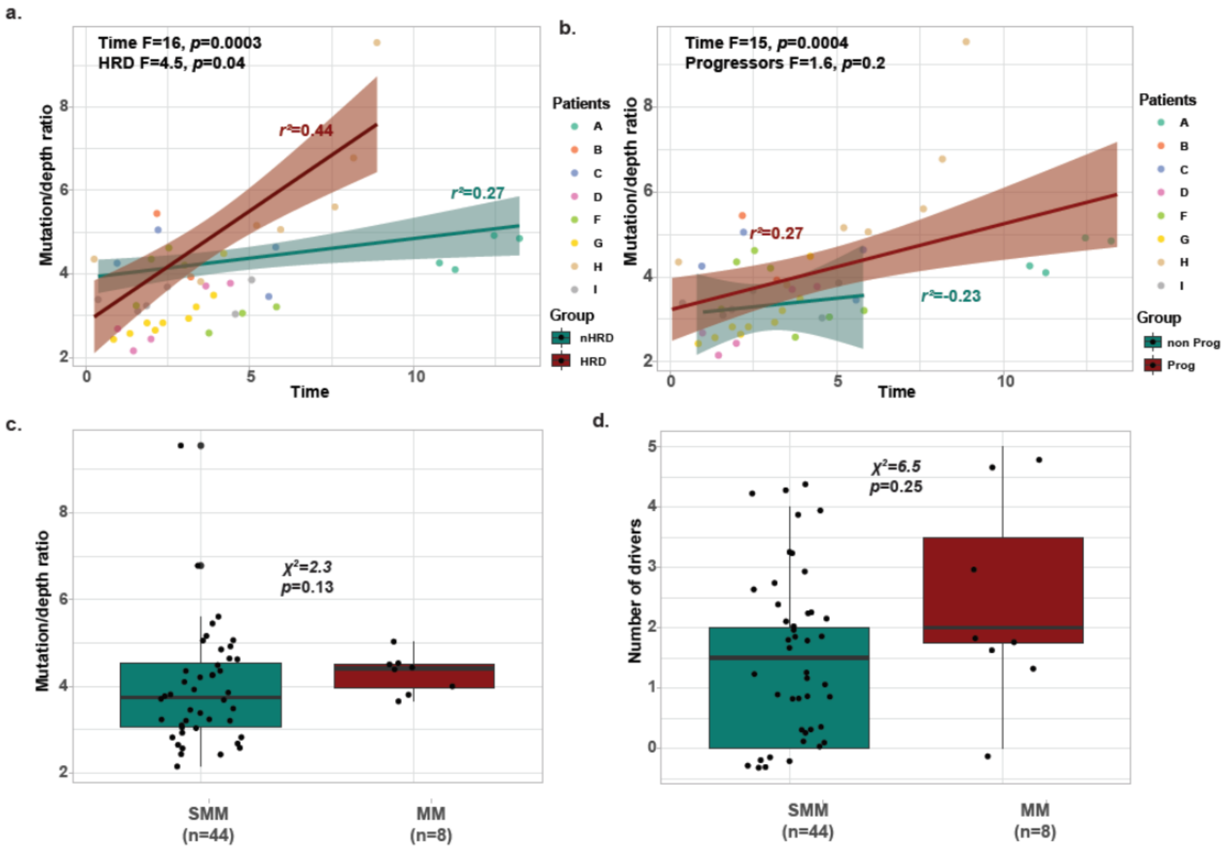

Supplemental Figure 10: Genomic evolution of Patient C. a. CCF plot showing the emergence of an *NF1* clone. b. Fishplot summarizing the clonal evolution in parallel to the paraprotein evolution. c. Phylogeny tree showing branching evolution.

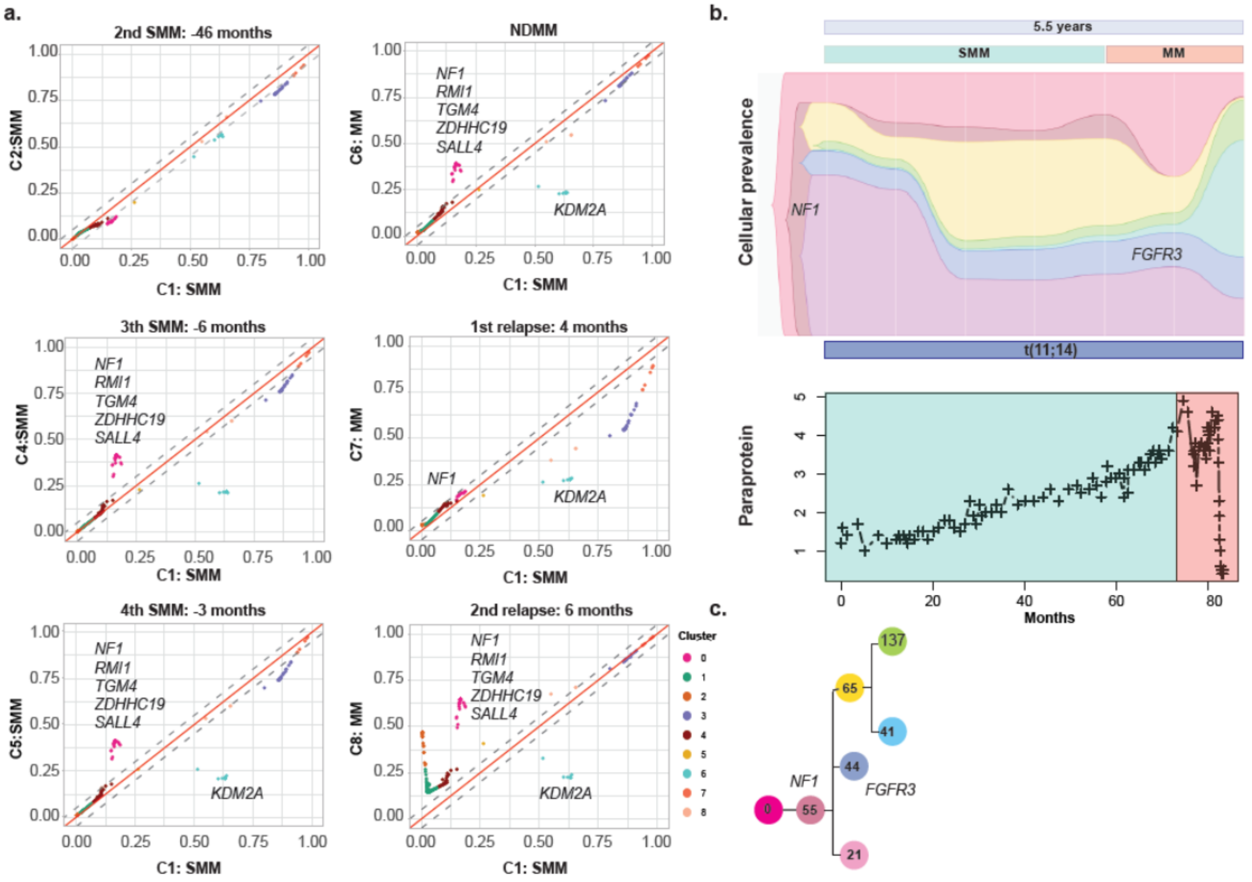

Supplemental Figure 11: Genomic evolution of Patient D. a. CCF plot showing the absence of clonal selection in a patient that has yet to progress. b. Fishplot summarizing the clonal evolution in parallel to the paraprotein evolution. c. Phylogeny tree showing branching evolution.

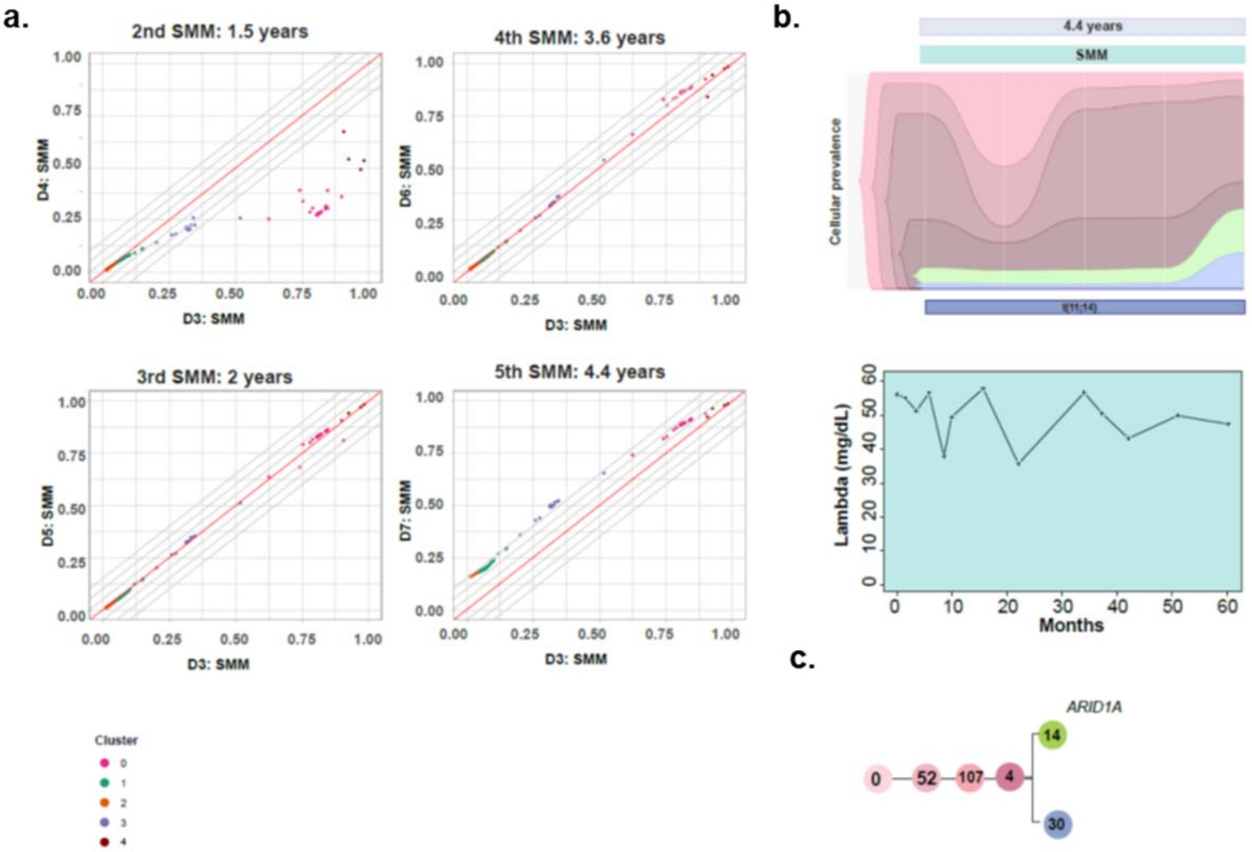

Supplemental Figure 12: Genomic evolution of Patient E. a. CCF plot showing the emergence of a *KRAS* and *MAPK6* clone. b. Fishplot summarizing the clonal evolution in parallel to the paraprotein evolution. c. Phylogeny tree showing branching evolution.

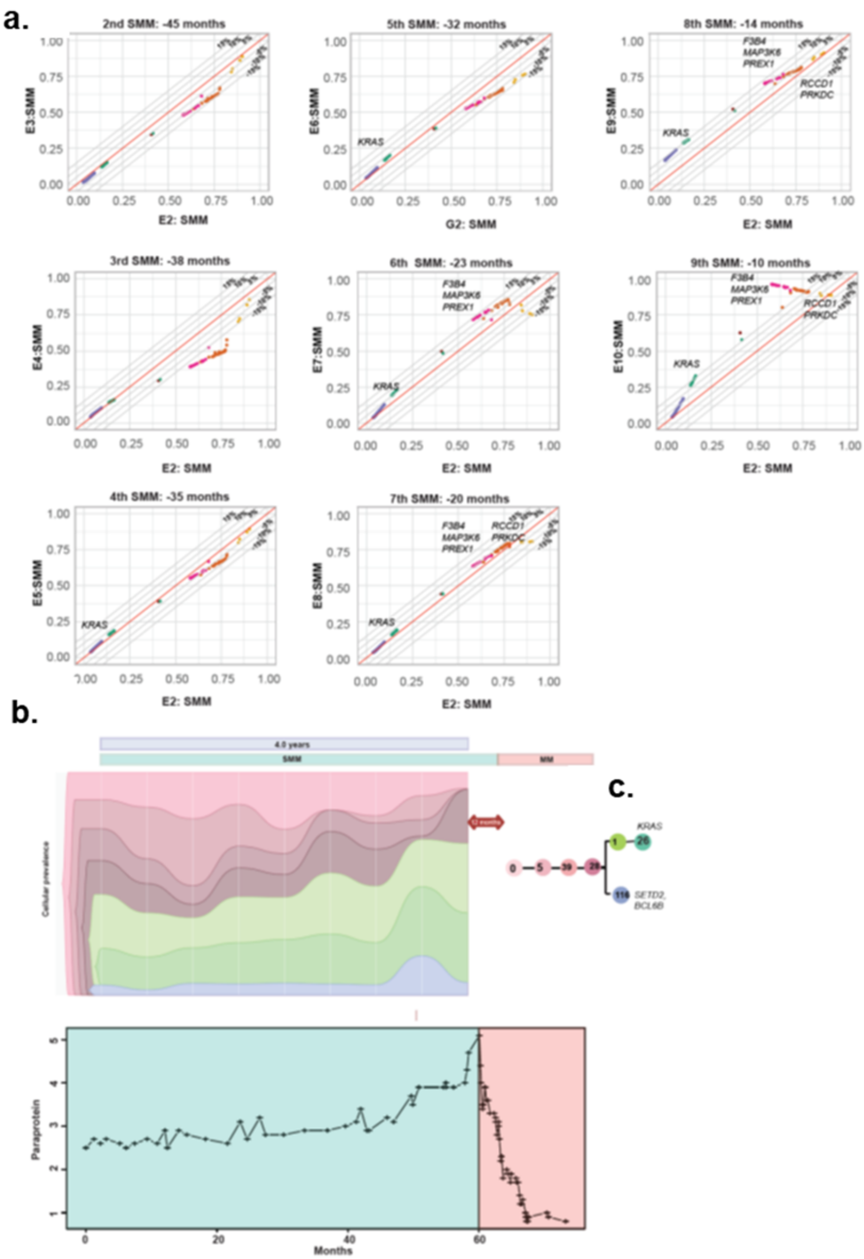

Supplemental Figure 13: Genomic evolution of Patient F. a. CCF plot showing stable clonal composition. b. Fishplot summarizing the clonal evolution in parallel to the paraprotein evolution. c. Phylogeny tree showing branching evolution.

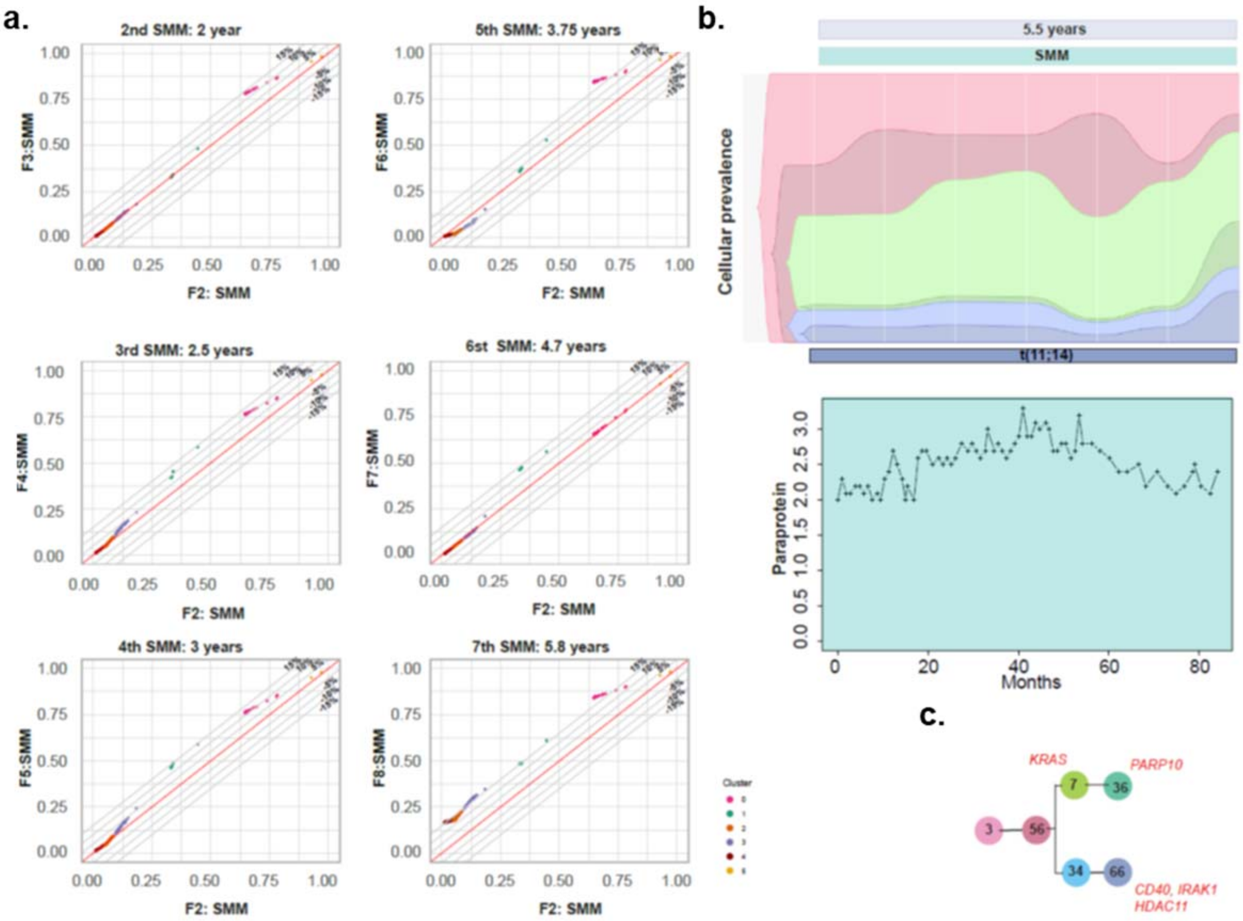

Supplemental Figure 14: Genomic evolution of Patient G. A. CCF plot showing the emergence of a *TNSFR1B* clone. B. Fishplot summarizing the clonal evolution in parallel to the paraprotein evolution. C. Phylogeny tree showing branching evolution

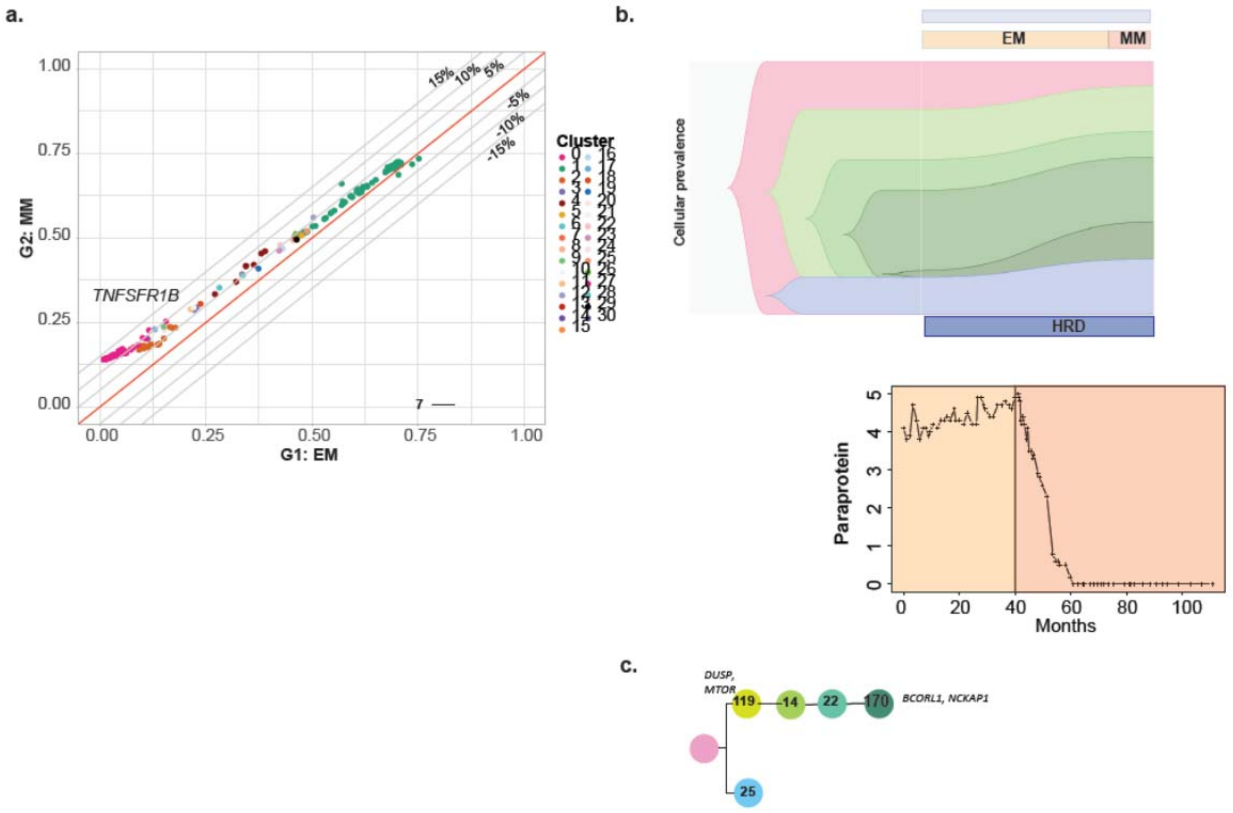

Supplemental Figure 15: Genomic evolution of Patient B. A. CCF plot showing the emergence of *ZFP36L1* clone. B. Fishplot summarising the clonal evolution in parallel to the paraprotein evolution. C. Phylogeny tree showing branching evolution

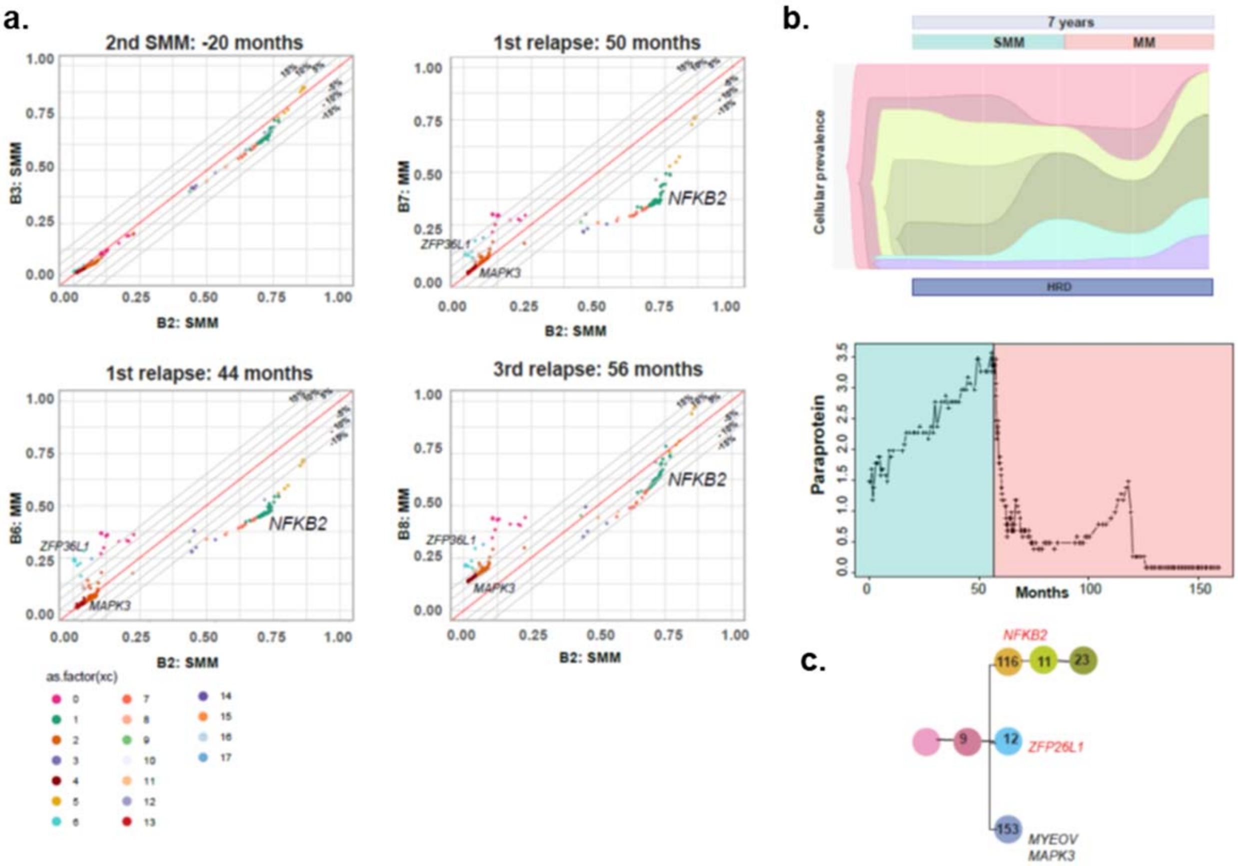

Supplemental Figure 16: Shannon diversity indices overtime. There was no linear correlation with time but patients that progressed had a stable H index whereas those that did not seemed to present changes in H index (a). Patients that progressed had significantly higher H indices than those that did not (b). There was no difference in H index between patients that had a *KRAS* mutation and those that did not (c). There was no difference in the H index of patients with a t(4;l14) and gain(1q) in comparison to those that did not (d) and there was no difference in H index between patients with either a t(4;14), gain(1q) or *KRAS* mutation in comparison to those that did not have any of these high risk features (e). Error bands 95% CI, n=number of patients, s=number of samples, Boxplot representing second quartile, median, and third quartile, whiskers representing first and last quartile, two-sided *p*-value derived from Kruskal-Wallis test

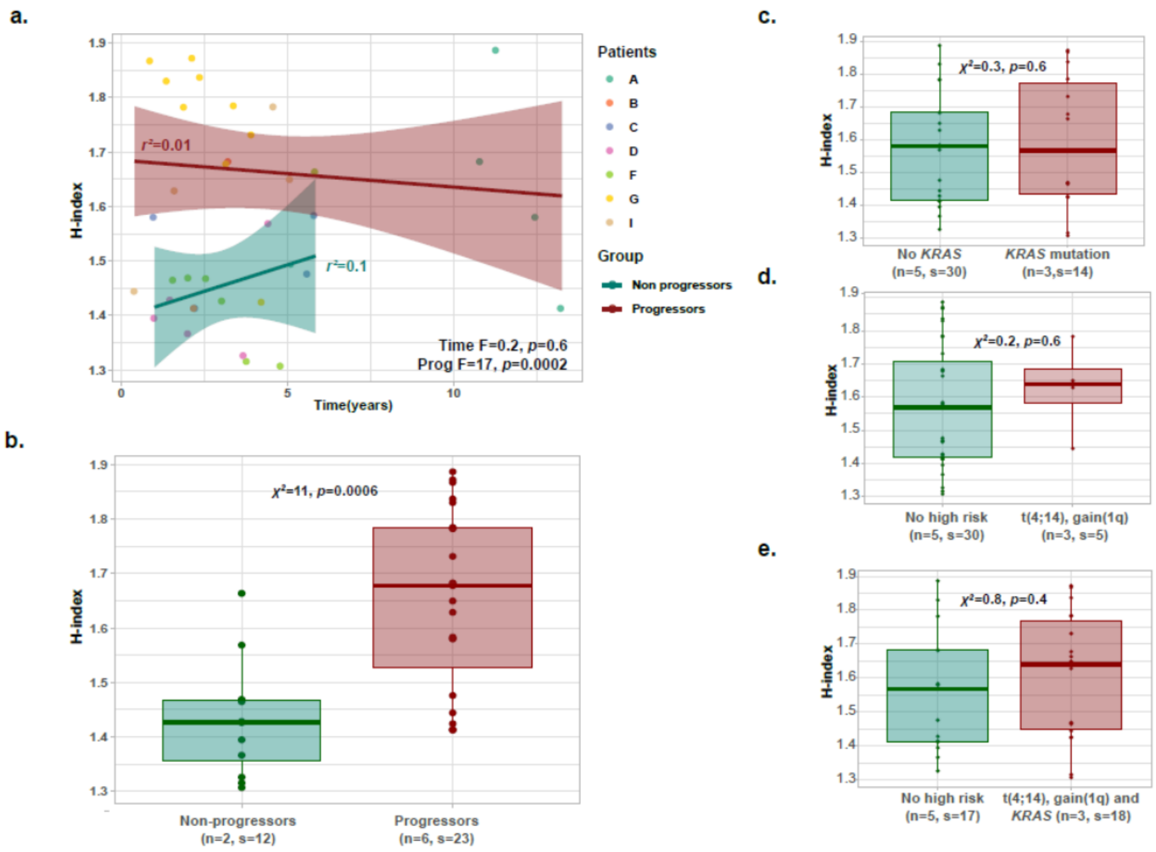

Supplement: Supplementary file 1 — Supplementary Information [file 41467_2020_20524_MOESM1_ESM.pdf]
