# Supplementary material for: The molecular make up of smoldering myeloma highlights the evolutionary pathways leading to multiple myeloma
Source: Nat Commun. 2021 Jan 12;12:293. doi: 10.1038/s41467-020-20524-2 (PMC7804406; doi:10.1038/s41467-020-20524-2)
Supplement: Supplementary file 3 — Reporting Summary [file 41467_2020_20524_MOESM3_ESM.pdf]

nature research

Corresponding author(s): Brian A Walker

Last updated by author(s): 10/072020

Reporting Summary

Nature Research wishes to improve the reproducibility of the work that we publish. This form provides structure for consistency and transparency in reporting. For further information on Nature Research policies, see our Editorial Policies and the Editorial Policy Checklist.

Statistics

For all statistical analyses, confirm that the following items are present in the figure legend, table legend, main text, or Methods section.

n/a	Confirmed	
☐	☒	The exact sample size (*n*) for each experimental group/condition, given as a discrete number and unit of measurement
☐	☒	A statement on whether measurements were taken from distinct samples or whether the same sample was measured repeatedly
☐	☒	The statistical test(s) used AND whether they are one- or two-sided<br>*Only common tests should be described solely by name; describe more complex techniques in the Methods section.*
☐	☒	A description of all covariates tested
☐	☒	A description of any assumptions or corrections, such as tests of normality and adjustment for multiple comparisons
☐	☒	A full description of the statistical parameters including central tendency (e.g. means) or other basic estimates (e.g. regression coefficient) AND variation (e.g. standard deviation) or associated estimates of uncertainty (e.g. confidence intervals)
☐	☒	For null hypothesis testing, the test statistic (e.g. *F*, *t*, *r*) with confidence intervals, effect sizes, degrees of freedom and *P* value noted<br>*Give P values as exact values whenever suitable.*
☐	☒	For Bayesian analysis, information on the choice of priors and Markov chain Monte Carlo settings
☒	☐	For hierarchical and complex designs, identification of the appropriate level for tests and full reporting of outcomes
☒	☐	Estimates of effect sizes (e.g. Cohen's *d*, Pearson's *r*), indicating how they were calculated

Our web collection on statistics for biologists contains articles on many of the points above.

Software and code

Policy information about availability of computer code

Data collection	Targeted panel: bcl2fastq was used for demultiplexing and BWA mem (v. 0.7.12) for alignment to Ensembl GRCh37/hg19 human reference genome. Strelka (v.1.0.14) was used for variant calling and single nucleotide variants (SNVs) were filtered using fpfilter (https://github.com/ckandoth/variant-filter). Indels were filtered using a 10% variant allele frequency (VAF) cut-off. Variants were annotated using Variant Effect Predictor (v.85). To determine copy number, a normalized depth comparison between tumor and control samples was used and segments of SNP variance were utilized to identify regions of chromosomal deletion and gain. Copy number was manually normalized based on the ratio and SNP allele calls using the best fitting chromosomes with the least variance (usually chromosome 2 or 10). Data were visualized using a custom built "RShiny" application showing the mutations, translocations, copy number, QC metrics and cross-sample contamination estimations, TarPan, available on GitHub (https://github.com/tcashby/tarpan) . Intra- and inter-chromosomal rearrangements were called using Manta (v0.29.6) with default settings and the exome flag specified.<br>Exome data: bcl2fastq was used for demultiplexing and BWA mem (v. 0.7.12) for alignment to Ensembl hg38 (exomes) human reference genome. Strelka (v.1.0.14) was used for variant calling and single nucleotide variants (SNVs) were filtered using fpfilter (https://github.com/ckandoth/variant-filter). Indels were filtered using a 10% variant allele frequency (VAF) cut-off. Variants were annotated using Variant Effect Predictor (v.85). Somatic copy-number aberration detection and tumor purity and ploidy estimation were performed using Sequenza v29 and log10 transformed ratio plotted using copynumber. Intra- and inter-chromosomal rearrangements were called using Manta (v0.29.6) with default settings and the exome flag specified.
Data analysis	Time-to-event analysis: Time-to-event analysis was performed in R with all genetic events with n>8. The Kaplan–Meier estimator was used to calculate time-to-event distributions. Stepwise Cox regression using well validated risk factors and potential novel factors.<br>Comparison testing: Kruskal-Wallis or Fisher's exact tests were used to compare the median of a continuous variable or the distribution of discrete variables across groups, when appropriate. Young's correction was used when appropriate. All p-values are two-sided if not specified otherwise.<br>Signature analysis

The fitting algorithm mmSig which fits the entire mutational catalogue of each patient with the mutational signatures involved in MM pathogenesis was used to determine the signature admixture in each individual sample and among samples more than two years away from progression, samples that were within two years of progression, and samples of patients that had not progressed. The contribution of each mutational signature was then corrected based on the cosine similarity between the original 96-mutational profile and the reconstructed profile generated without that signature.

For manuscripts utilizing custom algorithms or software that are central to the research but not yet described in published literature, software must be made available to editors and reviewers. We strongly encourage code deposition in a community repository (e.g. GitHub). See the Nature Research guidelines for submitting code & software for further information.

Data

Policy information about availability of data

All manuscripts must include a data availability statement. This statement should provide the following information, where applicable:

- Accession codes, unique identifiers, or web links for publicly available datasets
- A list of figures that have associated raw data
- A description of any restrictions on data availability

Theses data have been submited to EGA under accession numbers EGAD00001005056 (timeline project) and EGAD00001005285 (targeted panel).

Field-specific reporting

Please select the one below that is the best fit for your research. If you are not sure, read the appropriate sections before making your selection.

[x] Life sciences [ ] Behavioural & social sciences [ ] Ecological, evolutionary & environmental sciences

For a reference copy of the document with all sections, see nature.com/documents/nr-reporting-summary-flat.pdf

Life sciences study design

All studies must disclose on these points even when the disclosure is negative.

Sample size	All samples with sufficient material were analyzed.
Data exclusions	Patients that had been treated before progression to symptomatic myeloma were excluded from the PFS analysis as treatment may have affected their progression to symptomatic myeloma.
Replication	As SMM is a rare disease, especially sequential samples there are no replication study
Randomization	This is a retrospective observational study. No randomization was required.
Blinding	This is a retrospective observational study. No blinding was required.

Reporting for specific materials, systems and methods

We require information from authors about some types of materials, experimental systems and methods used in many studies. Here, indicate whether each material, system or method listed is relevant to your study. If you are not sure if a list item applies to your research, read the appropriate section before selecting a response.

Materials & experimental systems

n/a	Involved in the study	
[x]	[ ]	Antibodies
[x]	[ ]	Eukaryotic cell lines
[x]	[ ]	Palaeontology and archaeology
[x]	[ ]	Animals and other organisms
[ ]	[x]	Human research participants
[x]	[ ]	Clinical data
[x]	[ ]	Dual use research of concern

Methods

n/a	Involved in the study	
[x]	[ ]	ChIP-seq
[x]	[ ]	Flow cytometry
[x]	[ ]	MRI-based neuroimaging

Human research participants

Policy information about studies involving human research participants

Population characteristics	Eighty-two previously untreated SMM patients according to IMWG 2014 criteria were included in the study from the University of Arkansas for Medical Science after informed consent. The median follow-up was 5.18 years (95%CI 3.53-6.59) from diagnosis. Clinical data were collected and checked for consistency. A Ten early MM (EM) patients with new early myeloma criteria only (bone marrow plasma cell ≥60%, SFLC ratio ≥100, or more than one Focal lesion on MRI), and 17 MGUS were used for additional comparison. Additionally, 9 patients with repeated bone marrows were recruited. All patients had basic demographics (age, sex, race/ethnicity), clinical and biological data available.
Recruitment	Patients were recruited prospectively and samples bio-banked, on an observational study, which prospectively recruited patients with pre-malignant conditions such as MGUS, SMM or EM after informed consent UAMS MI 2012-12. Patients with sufficient available material were included in this study. This was a monocentric study, most samples being collected in the last 5 years.
Ethics oversight	This study was approved by the Institutional Review Boars (IRB) of the university of Arkansas for Medical Science (#261281). All research was conducted in accordance with the Declaration of Helsinki.

Note that full information on the approval of the study protocol must also be provided in the manuscript.
